# Genetic analysis and functional assessment of a *TGFBR2* variant in micrognathia and cleft palate

**DOI:** 10.1371/journal.pone.0324803

**Published:** 2025-06-09

**Authors:** JES-Rite Michaels, Paul P. R. Iyyanar, Ammar Husami, Andrew M. Vontell, Samantha A. Brugmann, Rolf W. Stottmann

**Affiliations:** 1 Steve and Cindy Rasmussen Institute for Genomic Medicine, Abigail Wexner Research Institute, Nationwide Children’s Hospital, Columbus, Ohio, United States of America; 2 Division of Human Genetics, Cincinnati Children’s Hospital Medical Center, Cincinnati, Ohio, United States of America; 3 Department of Pediatrics, University of Cincinnati College of Medicine, Cincinnati, Ohio, United States of America; 4 Division of Developmental Biology, Cincinnati Children’s Hospital Medical Center, Cincinnati, Ohio, United States of America; 5 Department of Pediatrics, School of Medicine, The Ohio State University, Columbus, Ohio, United States of America; Ohio State University, UNITED STATES OF AMERICA

## Abstract

Cleft lip and cleft palate are among the most common congenital anomalies and are the result of incomplete fusion of embryonic craniofacial processes or palatal shelves, respectively. We know that genetics play a large role in these anomalies but the list of known causal genes is far from complete. As part of a larger sequencing effort of patients with congenital craniofacial anomalies, we identified a rare candidate variant in *transforming growth factor beta receptor 2* (*TGFBR2*). This variant alters a highly conserved amino acid and is predicted to be pathogenic by a number of metrics. The family history and population genetics suggest that this specific variant would be incompletely penetrant, but this gene has been convincingly implicated in craniofacial development. In order to test the hypothesis this might be a causal variant, we used genome editing to create the orthologous variant in a new mouse model. Surprisingly, *Tgfbr2*^*V387M*^ mice did not exhibit craniofacial anomalies or have reduced survival, suggesting *Tgfbr2*^*V387M*^ is not a causal variant for cleft palate/ micrognathia. The discrepancy between in silico predictions and mouse phenotypes highlights the complexity of translating human genetic findings to mouse models. We expect these findings will aid in interpretation of future variants seen in *TGFBR2* from ongoing sequencing of patients with congenital craniofacial anomalies.

## Introduction

Cleft lip/cleft palate are craniofacial congenital anomalies characterized by incomplete fusion of the lip and/or palate during embryonic development. These are the among the most common of all congenital anomalies with an incidence of approximately 1/700 live births [1,2]. Causes of cleft lip and cleft palate are quite diverse with known contributions from genetics and environmental influences (teratogens) as well as the interplay between each [3,4]. Genetic studies of orofacial clefting disorders in humans have identified a handful of genes known to have high association with variable forms of cleft lip/ palate such as *IRF6* [5], *FOXE1* [6], and *TP63* [7]. These studies to date have been plagued with incomplete penetrance complicating a complete understanding of cleft lip/palate genetics [8,9]. We have been sequencing families with syndromic cleft lip/palate but no known genetic diagnosis to continue to build our knowledge of genetic variants leading to these conditions and potentially design future treatments.

Transforming Growth Factor beta (TGF-β) signaling is a classic developmental signaling pathway with extracellular ligands binding to complexes of transmembrane receptor tyrosine kinases. Ligand binding facilitates phosphorylation of type II receptors which then phosphorylates intracellular SMAD proteins such as SMAD2 to ultimately traffic to the nucleus and direct transcription of direct targets [10–13]. TGF-β signaling mediates several key functions during embryonic development including cell proliferation, differentiation, and extracellular matrix formation [14]. TGF superfamily signaling has strongly been associated with craniofacial development [15,16]. While the *transforming growth factor beta receptor 2* (*Tgfbr2*) null mouse embryos die by E10.5 [17], the role of *Tgfbr2* in craniofacial development became clear with studies using conditional inactivation in the cranial neural crest cells using the *Wnt1-Cre* driver. This ablation of *Tgfbr2* results in cleft palate due to reduced cell proliferation in the palatal mesenchyme at E14.5 [15,18–22]. In addition, these neural crest specific *Tgfbr2* mutants exhibited smaller mandibles with reduced condylar and coronoid processes and a complete lack of the angular process of the mandible in the proximal region [18,23]. Herein, we utilized the power of mouse genetics and precision genome editing to directly test if a candidate human variant in *TGFBR2* was a new causal variant for isolated micrognathia and cleft palate.

## Results

### A potentially pathogenic *TGFBR2* variant involved in craniofacial anomalies

**Fig 1:**
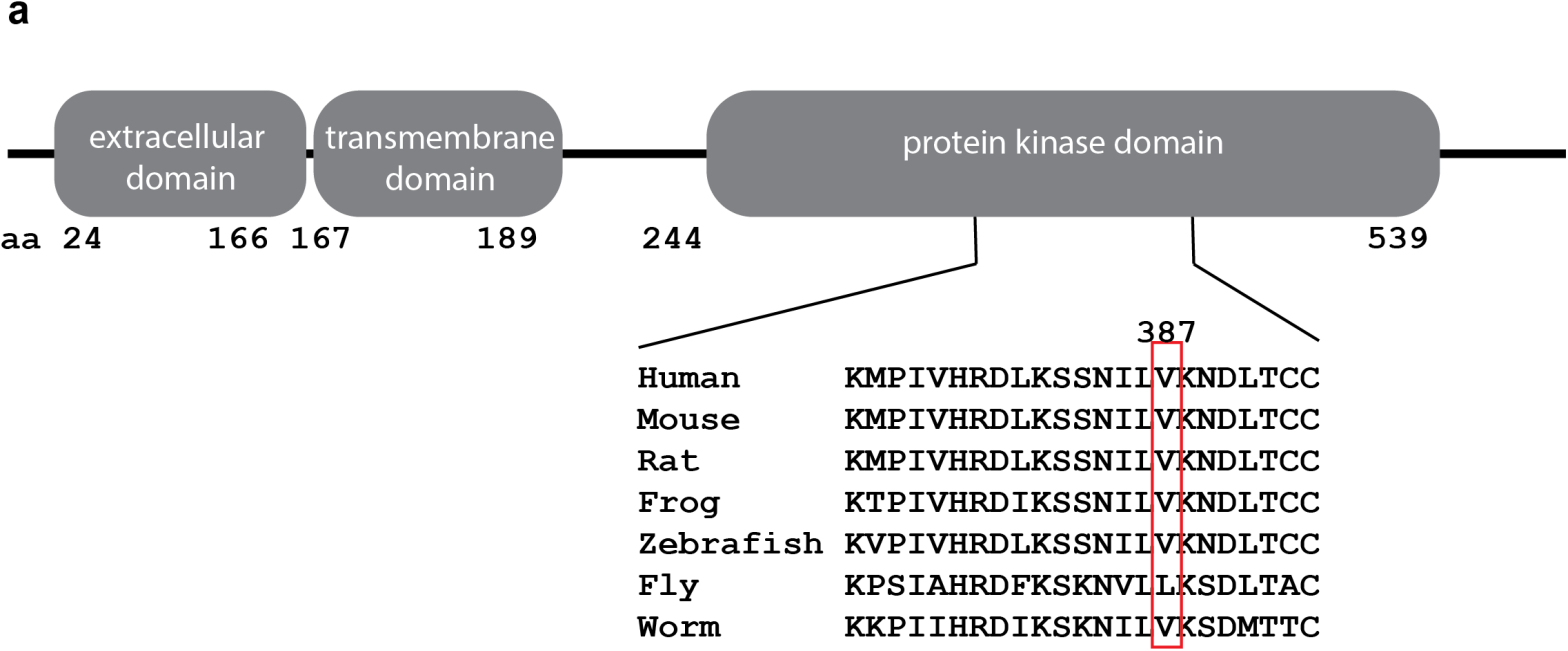
A novel *TGFBR2* allele. (a) TGFBR2 protein domain structure. Amino acid V387 is in an area of highly conserved sequence in the kinase domain.

We performed whole genome sequencing as part of a larger project on the human genetics of cleft lip/palate phenotypes. One recruited family had two affected children with cleft palate and micrognathia from unaffected parents with a previous maternal family history of cleft palate. No other significant phenotypes were noted in the affected children. Standard genome sequence variant analysis for rare variants in the affected children identified a candidate variant in *transforming growth factor beta receptor 2* (*TGFBR2*, NCBI Gene ID: 7048) which was present in both affected children and the unaffected mother. This variant (NM_003242.5:c.1159G > A; Chr3(GRCh37):g.30713824G > A; p.Val387Met) changes the amino acid at position 387 from a valine to a methionine. This variant has been noted previously (rs35766612) and is present at extremely low levels in control population databases with an overall allele frequency of 0.0017 and 4 homozygotes in gnomAD v4 [24], a virtually identical allele frequency of 0.0017 in the Regeneron million exome collection, and a combined annotation dependent depletion (CADD: [25] score of 24.4). The nucleotide sequence in this region of the genome is moderately conserved across phylogeny, but the amino acid code is faithfully conserved except for the *D. melanogaster* genome (Fig 1a). The change from valine to methionine only has a small physicochemical distance with a Grantham score of 21 (0–215) but is in the protein kinase domain which is critical for TGFBR2 function [26]. The variant is predicted to be “deleterious” by SIFT [27] (score = 0), “disease causing” by MutationTaster [28] (prob = 1), “probably damaging” by PolyPhen-2 [29] (score = 0.996), and “intolerant” by MetaDome [30] (score = 0.27). Previous reports in ClinVar [31] have conflicting interpretations of pathogenicity but were evaluated in different pathological contexts such as Marfan syndrome, Loeys-Dietz syndrome and aortic disease. We therefore hypothesized that this variant may be a cause of incompletely penetrant cleft palate and micrognathia in humans.

### A novel mouse model of *Tgfbr2*^*V387M*^

**Fig 2:**
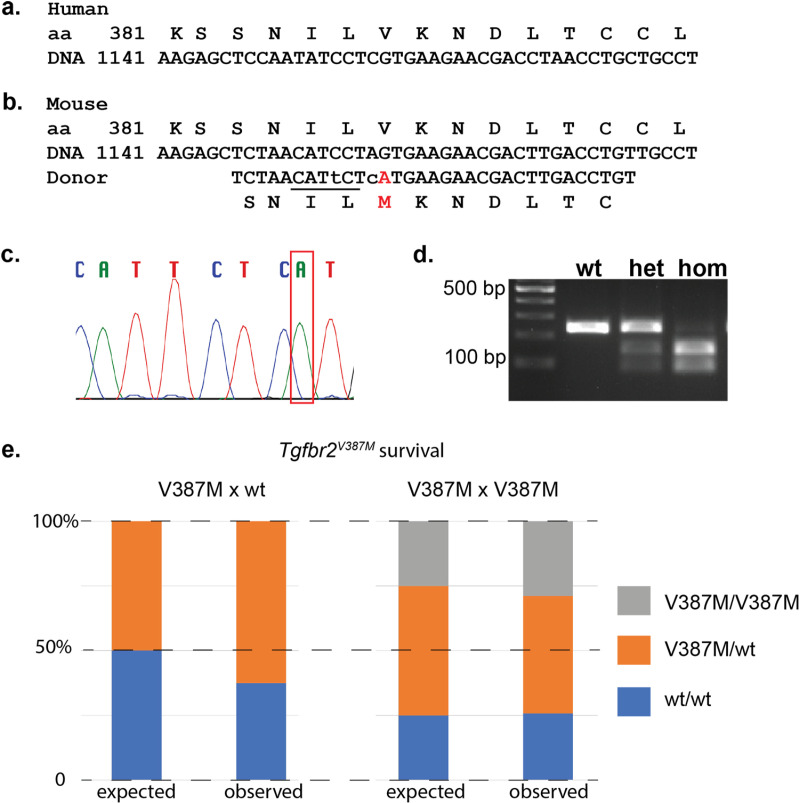
A mouse model of *Tgfbr2*^*V387M*^ missense allele. The amino acid and DNA sequence of *Tgfbr2* shows high conservation between human (a) and mouse (b). (c) Sanger sequence of mice showing desired sequence change from G to A indicated by the red box in mutants. (d) PCR genotyping followed by restriction digest indicating ability to clearly differentiate wild-type, heterozygous, and homozygous mutant mice. (e) Survival of all mice are not significantly different than Mendelian expectations.

Given the known role for *Tgfbr2* in craniofacial development and the incomplete penetrance of many craniofacial genetic variants in human, we created a mouse model to test the pathogenicity of this missense variant. This portion of the TGFBR2 protein is highly conserved between human (Fig 2a) and mouse (Fig 2b). CRISPR-CAS9 genome editing was used in one cell mouse embryos with injection of a mixture containing *Cas9*-gRNA and both variant knock-in and silent wild-type donors. Multiple founders were identified with the desired knock-in with Sanger sequencing and were bred to wild-type females. Animals from this outcross and their progeny were used in the analysis reported here. We designate this allele as *Tgfbr2*^*em1Rstot*^ but refer to it hereafter as *Tgfbr2*^*V387M*^. Genotyping was performed with a combination of Sanger sequencing (mutant allele shown in Fig 2c) and PCR followed by restriction digest as a variant in the donor oligonucleotide created a BspH1 recognition site (Fig 2d).

### *Tgfbr2*^*V387M*^ variant does not cause any craniofacial phenotypes

**Fig 3:**
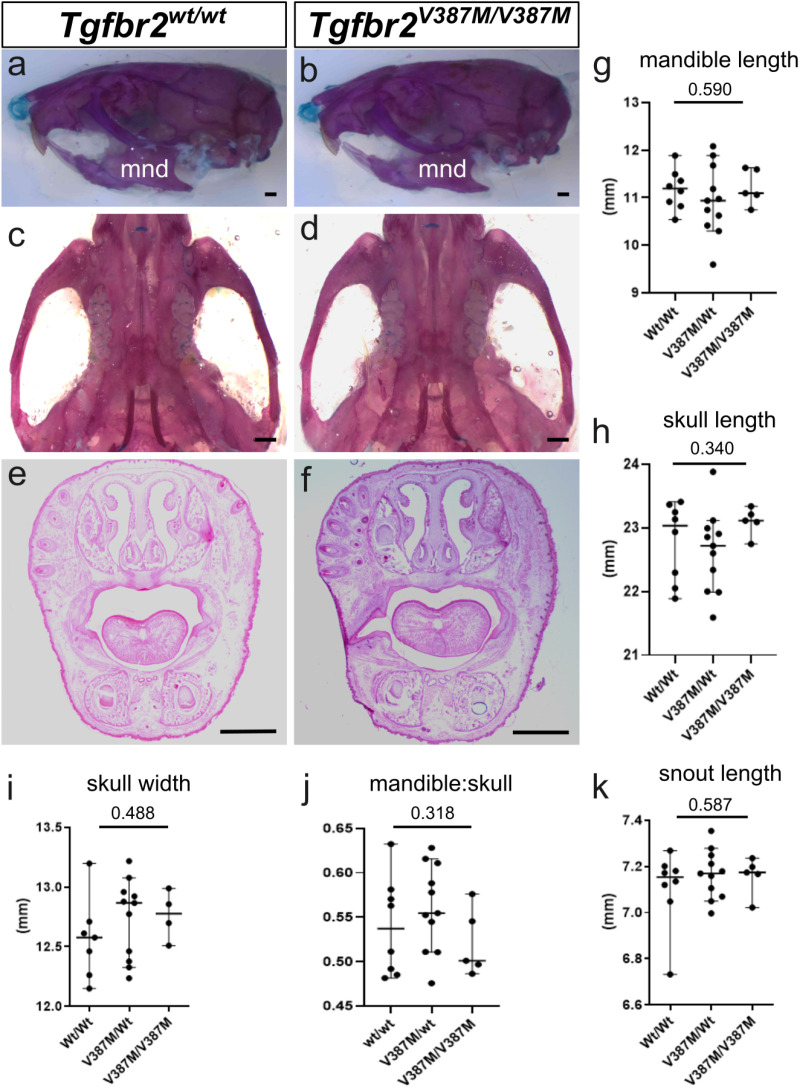
Craniofacial analysis of *Tgfbr2*^*V387M*^ mice. **(a-D)** Skeletal preparations of animals at postnatal day (P) 60 from the lateral view (a,b) or focused on the palatal surface (c,d) do not appear any different between wild-type (a,c) and *Tgfbr2* ^*V387M/V387M*^ homozygous mutants **(b,d)**. **(e,f)** Histological analysis of wild-types (e) and mutants (f) at E18.5 also did not reveal any differences. Quantification of mandible length **(g)**, skull length **(h)**, skull width **(i)**, ratio of mandible length to skull (j) or snout length (k) showed no difference between genotypes. Scale bars in a-f indicate 1 mm. Statistical values shown are from a one-way ANOVA.

We first analyzed survival of *Tgfbr2*^*V387M/wt*^ heterozygotes from crosses with wild-type animals and saw no reduced recovery of heterozygotes (n = 20 from 32 total animals; Chi-squared p-value = 0.157; Fig 2e, Table 1). To test if homozygotes have a phenotype, we intercrossed heterozygotes and saw no deviation from expected ratios of *Tgfbr2*^*V387M/wt*^ heterozygotes or *Tgfbr2*^*V387M/V387M*^ homozygotes from 66 weaned animals (Chi-squared p-value = 0.716; Fig 2e, Table 1). Cleft palate was not observed in any animals during the course of this study. Given that cleft palate is perinatal lethal in mouse models, we interpret these findings to suggest that cleft palate was very rarely, if ever, present in animals carrying this predicted pathogenic variant. One mechanism commonly resulting in cleft palate is obstruction of palatal shelf elevation and/or closure due to the posterior displacement of the tongue in micrognathia [32]. While this was not severe enough to cause cleft palate in these animals, if present at all, we tested if mutants may have micrognathia as suggested by the human participants. We performed skeletal preparations of animals born from *Tgfbr2*^*V387M/wt*^ heterozygous intercrosses to highlight cartilage and bone and measured the length of the mandible as well as the total head length (Fig 3a and 3b). Analysis did not show any reduction between genotypes in mandible length as an isolated measurement, or relative to skull length, at postnatal day (P)120 (Fig 3g–3k, n = 24). We also examined the roof of the mouth for any subtle deficits such as abnormal rugae or high arched palate, and noted no abnormalities (Fig 3c and 3d). We additionally performed histological analysis of a number of *Tgfbr2*^*V387M/V387M*^ homozygous mutants at E18.5 and P0 and did not observe craniofacial phenotypes in these animals either (Fig 3e and 3f). Finally, to assess if any subtle craniofacial abnormalities are present, we measured the skull length, skull width, and snout length in P120 head skeletal preparations. None of the aforementioned measurements in *Tgfbr2*^*V387M/wt*^ heterozygous or *Tgfbr2*^*V387M/V387M*^ homozygous heads were significantly different from wild-type heads (Fig 3h–3k). Thus, we conclude that the *Tgfbr2*^*V387M*^ missense variant did not lead to any craniofacial phenotypes in mouse.

**Table 1:** Survival of *Tgfbr2*^*V387M*^ animals at weaning.

	Total	wt/wt	V387M/ wt	V387M/V387M	Chi-sq. p value
**V387M x wt**					
expected	32	16	16	–	
observed	32	12	20	–	0.157
**V387M x V387M**					
expected	66	16.5	33	16.5	
observed	66	17	30	19	0.716

#### *Tgfbr2*^*V387M*^ variant does not lead *to* alterations *in* Smad phosphorylation levels.

**Fig 4:**
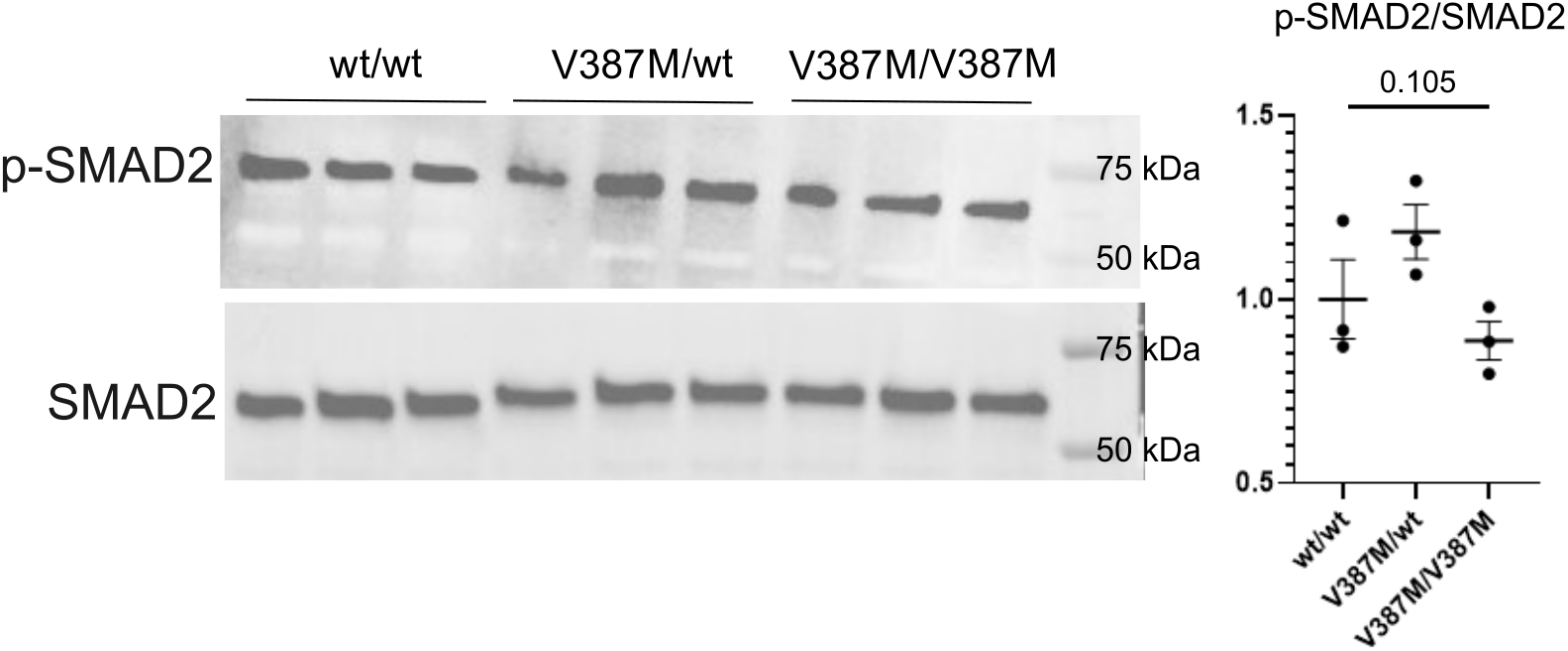
Western blot analysis of *TGF β* signaling in *Tgfbr2^V387M^* mice. Western blot analysis with antibodies for phosphorylated SMAD2 (p-SMAD2), and SMAD2 antibodies. Quantification shows the ratio of pSMAD2 to total SMAD2 is not significantly different among genotypes. Statistical values shown are from one-way ANOVA.

Binding of TGFβ ligands to TGFβR2 leads to heterodimerization with TGFβR1, which in turn phosphorylates the receptor associated SMAD2, which is the predominant SMAD downstream of TGFβ signaling during craniofacial development [14,33]. We therefore investigated the phosphorylated levels of SMAD2 (p-SMAD2) in the mandibles of *Tgfbr2*^*V387M/wt*^ heterozygous or *Tgfbr2*^*V387M/V387M*^ homozygous animals compared to wildtype at E13.5 (Fig 4). Our analysis did not reveal any significant difference in ratio of p-SMAD2 levels to total SMAD-2 level in either class of mutant. These data together show that the *Tgfbr2*^*V387M*^ mutation did not alter the level of TGFβR2-dependent phosphorylation of target proteins, further consistent with our hypothesis that the variant is not pathogenic.

## Discussion

Here we present a novel missense variant mouse allele of *Tgfbr2* created to assess the hypothesis the variant is causing disrupted craniofacial development with cleft palate due to micrognathia (as part of a presentation of Pierre Robin Sequence). While this variant was predicted to be deleterious by multiple algorithms, our findings in the mouse do not support a biological role for this variant as we do not observe reduced survival of *Tgfbr2*^*V387M/wt*^ heterozygous or *Tgfbr2*^*V387M/V387M*^ homozygous animals and do not find any phenotypes reminiscent of human participants. Furthermore, we do not see molecular evidence of disrupted TGFβ signaling. We conclude this is not likely to be a causal variant in human genetics but recognize this might be a failure of the mouse model to accurately recapitulate human biology.

### *TGFBR2* has known roles in human biology

Heterozygous missense variants in *TGFBR2* are well established to cause Loeys-Dietz Syndrome [34,35]. While cleft palate can be seen in some of these patients (two of the first six *TGFBR2* participants reported), the more common clinical characteristics include arterial aneurysm/dissection, skeletal phenotypes such as scoliosis or joint laxity, and craniosynostosis. The patients reported here were not known to have any clinical characteristics of Loeys-Dietz Syndrome and this particular variant we find has been most often classified as (likely) benign when analyzed in this context by clinical geneticists.

### *Tgfbr2* in craniofacial biology

TGFβ signaling plays multiple critical roles in cell proliferation, apoptosis, differentiation, and epithelial mesenchymal transition during embryonic development [14]. The binding of ligands (TGFβ1, TGFβ2 or TGFβ3) to the TGFBR2 receptor initiates the heterodimeric binding of TGFBR1. This heterodimerization leads to the phosphorylation of TGFBR1, which results in the phosphorylation of receptor associated SMADs such as SMAD2 and/or SMAD3 depending on the context [14,15]. Among the mouse orthologues of TGFβ ligands, null mutants of *Tgf*β*2* [36]and *Tgf*β*3* [37,38] develop cleft palate with a penetrance of 23% and 100%, respectively. *Tgf*β*3* is exclusively expressed in the palatal epithelia including the medial edge epithelia and midline epithelial seam during palatal fusion and is critical for p-SMAD2 activation, but not p-SMAD3 [39]. *Smad2* is critical for embryonic development as *Smad2*^*-/-*^ embryos die by E8.5, while *Smad3*^*-/-*^ mice survive postnatally [33,40]. Interestingly, some of the *Smad2* heterozygous mice develop severe mandibular hypoplasia with proboscis-like phenotype [33].

Since *Tgfbr2* homozygous null embryos die by E10.5 [17], a *Wnt1*-Cre mediated deletion was employed to gain additional information regarding the role of *Tgfbr2* in craniofacial development [18]. *Wnt1-Cre;Tgfbr2*^*flox/flox*^ mutants have a completely penetrant complete failure of fusion in the secondary palate, likely due to reduced proliferation of the neural crest cells as they facilitate horizontal growth of the palatal shelves during mid-gestation (E14.5) [15,18–22]. This neural crest specific ablation of *Tgfbr2* results in an increase in the mRNA expression of *Tgf*β*2* and *Tgfbr3*. In these mutants, upregulated TGFβ2 binds and signals through TGFBR3 to activate non-canonical TGFβ signaling via p38 MAPK signaling resulting in defective cell proliferation [19]. Interestingly, the cleft palate phenotype of *Tgfbr2* mutants is rescued by the haploinsufficiency of *Tgf*β*2* (82.5%) or *Tgfbr1* (91.7%) [19]. These findings further highlight the intricacy of the unique roles played by individual TGFβ ligands and receptors during normal development and in the pathogenesis of craniofacial malformations. Our data on the *Tgfbr*2^*V387M*^ mutation revealed that levels of p-SMAD2, a critical downstream regulator of the TGFβ signaling, was not affected in these mutants, implying that the mutation does not affect receptor-dependent downstream signaling. This is understandable given the lack of craniofacial phenotype in these mutants. In addition, neural crest-specific inactivation of *Tgfbr2* resulted in a hypoplastic mandible with severe defects in the proximal region [18,23]. This conditional ablation of *Tgfbr2* also leads to skull defects which are also seen in Loeys-Dietz Syndrome patients. Neither the mouse model we report here, nor the sequenced family, show any evidence for craniosynostosis or defects of the calvaria.

#### Mouse as a model for human congenital craniofacial anomalies.

Genetic similarities, experimental tools and overall conserved processes of development and disease between mouse and humans have supported the rise of the mouse as the most powerful mammalian model of human development and disease. The power in this approach has only grown with the advent of CRISPR-CAS9 mediated genome editing and the ability to manipulate the genome to model specific variants seen in human patients. Thus, we took this approach with the aim of assessing pathogenicity of a specific variant identified through whole genome sequencing. In addition to addressing specific hypotheses about genetic causes of human disease, these mouse models can facilitate experiments to determine underlying molecular mechanism(s) and potentially test proof of concept experiments for therapeutic interventions.

One alternative explanation for our data is that the *Tgfbr2*^*V387M*^ variant is a risk factor for cleft lip/palate in humans, but this is not recapitulated in the mouse due to subtle differences in the two models. While the processes of craniofacial development broadly, and palatogenesis specifically, are quite similar, between mice and humans, there are some developmental differences [41,42]. In general, however, there is concordance between the mouse and human model. A recent review compiled a list of mouse models with cleft palate and human disorders associated with those genes [43]. In this comparison, 15 human diseases with cleft palate as a phenotypic feature had a corresponding mouse models cataloged and all were noted to have cleft palate, including the *Tgfb2* ligand itself [36]. Another review found a similarly very high concordance [41]. Even in the biochemical pathway we are considering here, we have mentioned multiple mutants of the TGFβ signaling cascade which have cleft palate in mouse mutants [18,36].

Another variable to consider is genetic background. We conducted our experiments with mice on a C57BL/6J background. Mouse strains are known to have different sensitivities to clefting, including environmental influences [44], but the C57BL/6J mouse has not been previously shown to be particularly refractory to craniofacial differences. Indeed, the previously described conditional ablation of *Tgfbr2* in the neural crest cell lineage (*Wnt1-Cre; Tgfbr2*^*floxl/flox*^) was also in the C57BL/6J background [18], further reiterating that the background may not be the attributing factor for the lack of cleft palate phenotype in our analysis. A classic mouse model of Treacher Collins Syndrome is also more acutely affected in C57BL/6J as compared to the DBA strain [45]. Thus, it is more likely that this *Tgfbr2* variant is not a causal allele for cleft palate or micrognathia leading to palate disruption in mammals despite the deep conservation and *in silico* predicted pathogenicity. In this case, the population data was not consistent with a highly penetrant phenotype. These population frequency datasets are getting more powerful with the addition of new data and will therefore become increasingly valuable and predictive in future analyses. The ability to recapitulate human variants in a genetically tractable model like the mouse will continue to be an attractive experimental option in future studies of human disease genetics.

## Methods

### DNA sequencing

DNA was collected as part of an IRB-approved recruitment protocol and purified using the Qiagen DNeasy Kit and manufacturer’s protocols (Qiagen, USA). Whole genome sequencing was done at Novogene USA to an average depth of at least 30x with standard protocols.

### Variant discovery

VCF format small SNP and InDels were annotated and filtered using standardized protocols using GoldenHelix VarSeq and Qiagen – Qiagen Clinical Interpreter – Translational (formerly Ingenuity) variant analysis software packages. The variant annotation and interpretation analyses were generated through the use of Ingenuity® Variant Analysis™ software https://wwusingtics.com/products/ingenuity-variant-analysis from QIAGEN, Inc. Variants were filtered based on population frequencies. Subsequently, variants known or predicted to have semantic similarity with Micrognathia, and cleft palate were discovered using Phenotype Driven Ranking (PDR) filter.

### Mouse model creation

Mice (NCBI Taxon ID 10090) were created at the Cincinnati Children’s transgenic animal and genome editing core (RRID:SCR_022642). Mouse zygotes (C57BL/6N strain) were injected with 200 ng/μl CAS9 protein (IDT and ThermoFisher), 100 ng/μl *Tgfbr2*-specific sgRNA (AGGTCAAGTCGTTCTTCACT), 75 ng/μl single-stranded donor oligo- nucleotide (KI) to create the *Tgfbr2* variant (AGAGCTGGGCAAGCAGTACTGGCTGATCACGGCGTTCCACGCGAAGGGCAACCTGCAGGAGTACCTCACGAGGCATGTCATCAGCTGGGAGGACCTGAGGAAGCTGGGCAGCTCCCTGGCCCGGGGATCGCTCATCTCCACAGTGACCACACTCCTTGTGGGAGGCC; IDT, Iowa) and 75 ng/μl single-stranded donor oligo- nucleotide of wild-type sequence (WT-S) with silent variants (TGTTGGCCAGGTCATCCACAGACAGAGTAGGGTCCAGGCGCAAGGACAGCCCGAAGTCACACAGGCAACAGGTCAAGTCGTTCTTCAcgAGaATGTTAGAGCTCTTGAGGTCCCTGTGAACAATGGGCATCTTG; IDT, Iowa) followed by surgical implantation into pseudo-pregnant female (CD-1 strain) mice. Both donors are used together to prevent possible lethality of homozygous target mutations. Silent mutations (indicated by the lowercase letters in Fig 2b) are also introduced in the knock-in donor to prevent recutting of the target allele by CRISPR and to create a restriction enzyme site to facilitate genotyping. PCR genotyping was performed by amplification of genomic DNA (F:CATCGCTCATCTCCACAGTGAC and R:TGAAGCCAGGCATGAAGTCTGAG primers).

The PCR products were subject to digestion with BspH1 or Sanger Sequencing and pups exhibiting editing of interest were then crossed to wild-type (C57BL/6J) mice and the resulting progeny were Sanger sequenced (CCHMC DNA Sequencing and Genotyping Core) to confirm the alleles generated. Propagation of the *Tgfbr2* allele was done by crossing to wild-type C57BL/6J mice and/or intercross. Further genotyping was with a combination of PCR followed by restriction digest and/or Sanger sequencing.

### Animal housing

All experiments using mice in this study were performed using ethically acceptable procedures as approved by the Institutional Animal Care and Use Committee at Nationwide Children’s Hospital (AR21–00067). Mice were fed mouse breeder diet and housed in ventilated cages with a 12 h light/12 h dark cycle. Animals were euthanized with carbon dioxide asphyxiation consistent with standard practices. No anesthesia and/or analgesia methods were required and all efforts were made to alleviate animal suffering.

### Skeletal preps and pictures

Skeletons from P60 or P120 animals were stained with Alcian Blue and Alizarin Red to visualize cartilage and bone, respectively. Briefly, animals were skinned, eviscerated and fixed for 2 days in 95% ethanol. They were stained overnight at room temperature in 0.03% (w/v) Alcian Blue solution (Sigma- Aldrich, A3157) containing 80% ethanol and 20% glacial acetic acid. Samples were destained in 95% ethanol for 24 h followed by pre-clearing in 1% KOH overnight at room temperature. Skeletons were then stained overnight in 0.005% Alizarin Red solution (Sigma-Aldrich, A5533) containing 1% KOH. A second round of clearing was performed by incubating tissues in 20% glycerol/1% KOH solution for 24 h. Finally, they were transferred to 50% glycerol/50% ethanol for photography. Skeletal preparations were imaged using a Zeiss Discovery.V12 Stereoscope and length measurements were recorded for mandibular bones and skull length (tip of nasal bone to basioccipital bone). For skull width, measurements were made between the bilateral zygomatic processes at the third molar level. For snout length, measurements were made between the premaxilla to the anterior end of the first molar tooth.

### Histology

Hematoxylin & Eosin (H&E) fixation was in Bouin’s solution followed by washes in 70% ethanol. For H&E staining, embryos were embedded in paraffin and cut to 10 μm sections before staining using standard techniques (Behringer, 2014). All images were taken via Zeiss Discovery.V12 Stereoscope. Paired images are shown at the same magnification.

### Western blot analysis

Western blot analysis was carried out as previously described [46]. Briefly, mandibles from E13.5 *Tgfbr2*^*V387M/wt*^ heterozygous or *Tgfbr2*^*V387M/V387M*^ homozygous, and wildtype littermates were micro-dissected and homogenized in RIPA buffer with protease and phosphatase inhibitors (Pierce). Proteins were quantified using BCA assay (Pierce) and 20 µg of protein was separated on a 4–20% mini-Protean TGX stain-free gels (Bio-Rad). Proteins were transferred to 0.45 µm PVDF membrane by Turbo blot transfer and imaged using stain free imaging (Bio-Rad). Blots were blocked with Everyblot blocking buffer (Bio-Rad) for 10 minutes. Primary antibodies were p-SMAD2 (1:1000; Cell Signaling) and SMAD2 (1:1000; Cell Signaling). Blots were washed in 1X TBST and probed with anti-rabbit IgG-HRP conjugate (Abcam; 1:3000) and developed using Clarity ECL substrate (Bio-Rad). Western blot images were quantified using the AlphaView Software. Band intensities of p-SMAD2 or SMAD2 for each lane were normalized to their respective total protein levels from stain free total protein gel images. The relative intensities of p-SMAD2 were normalized to their SMAD2 levels and analyzed ordinary one-way ANOVA with Tukey’s multiple comparison post-hoc test.

### Statistics

All statistical analyses were performed using GraphPad Prism 9.5.1. Ordinary one-way ANOVA with Dunnett’s post-hoc multiple comparisons test was performed for comparison of mandible lengths. ANOVA P values are indicated on all graphs. The data shown are the median ± 95% confidence interval. No samples were excluded from analyses.

## Supporting information

S1 File XXX. (DOCX)
