# Supplementary material for: Genetic analysis and functional assessment of a TGFBR2 variant in micrognathia and cleft palate
Source: PLoS One. 2025 Jun 9;20(6):e0324803. doi: 10.1371/journal.pone.0324803 (PMC12148102; doi:10.1371/journal.pone.0324803)
Supplement: S1 File — (DOCX) [file pone.0324803.s001.docx]

SUPPORTING INFORMATION FILES

Original images for blot and gel results

Gel showing genotyping information as shown in Fig. 2d


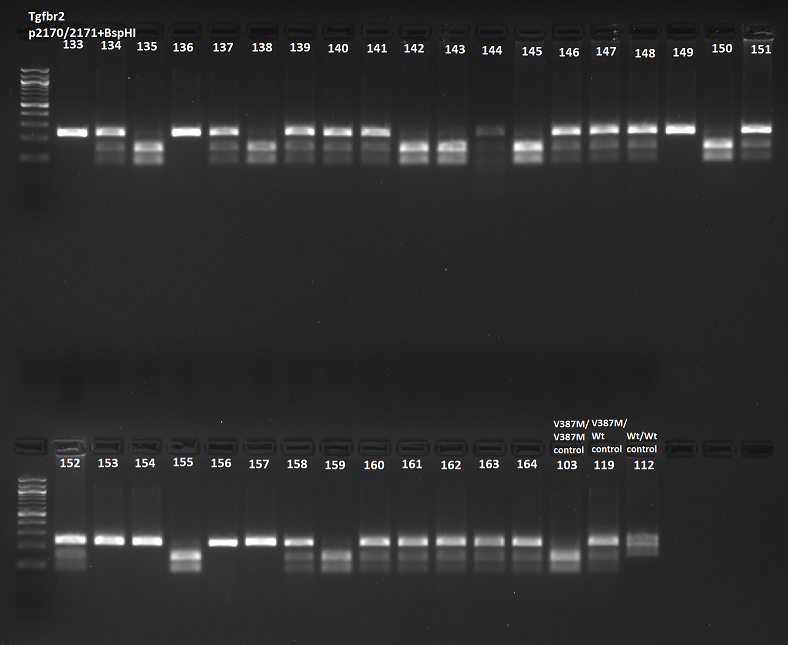


Immuno blots as used in Fig 4.

p-SMAD2 SMAD2


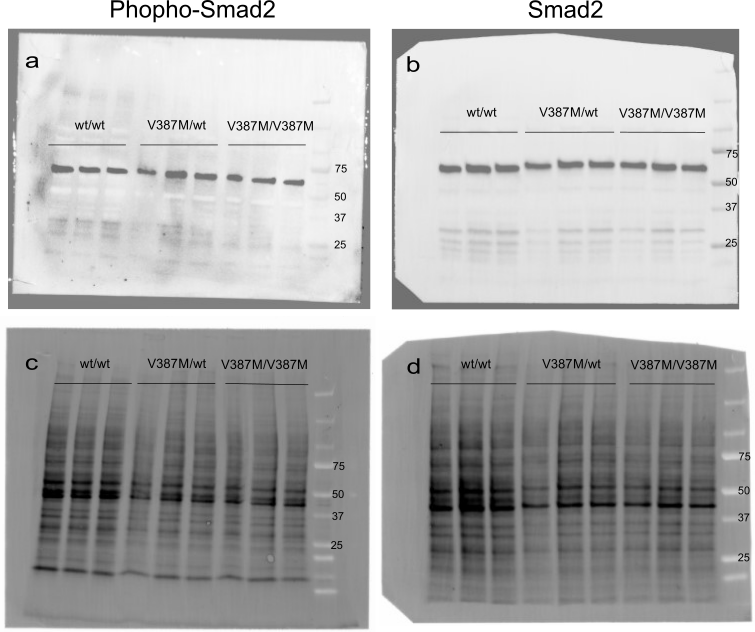


Antibody in a,b against pSMAD2 and SMAD2, respectively. Total protein stain for each in c,d.

Quantification of Western blots

| **Sample** | **pSmad2** | **Total protein** | **pSmad2/Total protein** | **pSmad2/ Total Smad2 after total protein normalization** | **Relative band intensity pSmad2/Total Smad2** |
| --- | --- | --- | --- | --- | --- |
| WT-1 | 91.35 | 182.66 | 0.500109493 | 0.977072609 | 1.213959496 |
| WT-2 | 71.61 | 173.77 | 0.412096449 | 0.736538835 | 0.915109383 |
| WT-3 | 66.78 | 171.84 | 0.388617318 | 0.70098133 | 0.87093112 |
| V387M/Wt | 75.45 | 148.6 | 0.507738896 | 0.858539195 | 1.066688185 |
| V387M/Wt | 97.41 | 159.48 | 0.610797592 | 1.064010666 | 1.321975297 |
| V387M/Wt | 82.93 | 156.67 | 0.529329163 | 0.934169823 | 1.160655122 |
| V387M/V387M | 65.4 | 158.13 | 0.41358376 | 0.712241412 | 0.884921159 |
| V387M/V387M | 70.28 | 151.69 | 0.463313336 | 0.787527373 | 0.978459865 |
| V387M/V387M | 59.84 | 156.85 | 0.381510998 | 0.641777162 | 0.797373167 |
|  |  |  |  |  |  |
|  |  |  |  |  |  |
|  |  | Avg in WT | 0.433607754 | 0.804864258 |  |
|  |  |  |  |  |  |
|  |  |  |  |  |  |
| **Sample** | **Total Smad2** | **Total protein** | **Smad2/Total protein** |  |  |
| WT-1 | 81.24 | 158.72 | 0.511844758 |  |  |
| WT-2 | 94.31 | 168.56 | 0.559504034 |  |  |
| WT-3 | 87.76 | 158.3 | 0.554390398 |  |  |
| V387M/Wt | 82.37 | 139.28 | 0.591398621 |  |  |
| V387M/Wt | 87.21 | 151.92 | 0.574052133 |  |  |
| V387M/Wt | 83.68 | 147.68 | 0.566630553 |  |  |
| V387M/V387M | 82.59 | 142.23 | 0.580679182 |  |  |
| V387M/V387M | 88 | 149.58 | 0.588313946 |  |  |
| V387M/V387M | 83.7 | 140.8 | 0.594460227 |  |  |
|  |  |  |  |  |  |
|  |  | Avg in WT | 0.541913063 |  |  |

All skeletal measurements

| **STRAIN** | **ANIMAL** | **Stage** | **MANDIBLE LENGTH (ML) µm** | **HEAD LENGTH (HL) µm** | **Head Width µm** | **Snout length µm** | **ML to HL Ratio (ML:HL)** | **Genotype** |
| --- | --- | --- | --- | --- | --- | --- | --- | --- |
| Tgfbr2 | 60 | p120 | 10913.242 | 22297.382 | 12461.073 | 7047.96 | 0.489440509 | Wt/Wt |
| Tgfbr2 | 61 | p120 | 11240.781 | 21888.377 | 12261.336 | 7135.289 | 0.513550228 | Wt/Wt |
| Tgfbr2 | 65 | p120 | 10536.213 | 22050.465 | 13197.597 | 7268.673 | 0.477822713 | Wt/Wt |
| Tgfbr2 | 69 | p120 | 10821.006 | 22936.811 | 12710.209 | 7200.855 | 0.471774651 | Wt/Wt |
| Tgfbr2 | 77 | p120 | 11883.649 | 23246.147 | 12576.746 | 6732.423 | 0.511209406 | Wt/Wt |
| Tgfbr2 | 80 | p120 | 11353.16 | 23407.865 | NA | 7180.685 | 0.485014759 | Wt/Wt |
| Tgfbr2 | 81 | p120 | 11492.436 | 23366.805 | 12149.71 | 7119.933 | 0.491827445 | Wt/Wt |
| Tgfbr2 | 83 | p120 | 11137.806 | 23132.753 | 12610.884 | 7172.136 | 0.481473433 | Wt/Wt |
| Tgfbr2 | 58 | p120 | 9593.768 | 22601.587 | 12866.628 | 7159.36 | 0.424473202 | V387M/Wt |
| Tgfbr2 | 62 | p120 | 11183.329 | 21592.195 | 12461.739 | 7069.329 | 0.517933865 | V387M/Wt |
| Tgfbr2 | 63 | p120 | 10740.438 | 22002.207 | 12327.108 | 7049.491 | 0.488152757 | V387M/Wt |
| Tgfbr2 | 64 | p120 | 10297.72 | 21987.496 | 13077.775 | 7354.029 | 0.468344372 | V387M/Wt |
| Tgfbr2 | 66 | p120 | 10972.906 | 22719.67 | 12877.704 | 7179.067 | 0.482969427 | V387M/Wt |
| Tgfbr2 | 68 | p120 | 12077.725 | 23881.731 | 13216.516 | 7248.238 | 0.505730719 | V387M/Wt |
| Tgfbr2 | 70 | p120 | 11885.315 | 23115.969 | 12921.791 | 7169.002 | 0.514160362 | V387M/Wt |
| Tgfbr2 | 71 | p120 | 10415.472 | 22906.1 | 12773.238 | 7105.698 | 0.454702983 | V387M/Wt |
| Tgfbr2 | 72 | p120 | 10623.869 | 22339.067 | 12958.335 | 7211.021 | 0.475573532 | V387M/Wt |
| Tgfbr2 | 84 | p120 | 11676.49 | 22857.376 | 12235.949 | 6996.838 | 0.510841227 | V387M/Wt |
| Tgfbr2 | 85 | p120 | 10935.134 | 22992.716 | 12377.046 | 7278.577 | 0.475591226 | V387M/Wt |
| Tgfbr2 | 59 | p120 | 10742.294 | 23112.965 | 12856.632 | 7173.927 | 0.464773516 | V387M/V387M |
| Tgfbr2 | 67 | p120 | 11093.855 | 23090.429 | 12988.041 | 7197.881 | 0.480452529 | V387M/V387M |
| Tgfbr2 | 78 | p120 | 11060.376 | 22745.14 | NA | 7021.972 | 0.486274255 | V387M/V387M |
| Tgfbr2 | 79 | p120 | 11592.658 | 23338.741 | 12697.045 | 7235.824 | 0.496713083 | V387M/V387M |
| Tgfbr2 | 82 | p120 | 11626.411 | 23211.91 | 12508.579 | 7166.945 | 0.500881272 | V387M/V387M |
